# Attachment Representation and Emotion Recognition Ability in Children with ADHD and Their Parents: A Study Protocol

**DOI:** 10.3390/ijerph18052277

**Published:** 2021-02-25

**Authors:** Ruediger Kissgen, Sebastian Franke, Moritz Susewind, Maya Krischer

**Affiliations:** 1 Developmental Science and Special Education, Department of Educational Sciences and Psychology, University of Siegen, 57068 Siegen, Germany; sebastian.franke@uni-siegen.de; 2 Department of Child and Adolescent Psychiatry, Psychosomatics and Psychotherapy, Medical Faculty, University of Cologne, 50931 Cologne, Germany; M.Susewind@ptz.de (M.S.); maya.krischer@uk-koeln.de (M.K.)

**Keywords:** attachment, ADHD, emotion recognition, school age, children, mothers, fathers

## Abstract

*Background*: Few studies in clinical attachment research to date have examined children with an attention-deficit/hyperactivity disorder (ADHD) diagnosis. This is surprising for two reasons: first, there are a number of parallels between the behaviors of children with an insecure and disorganized attachment and the behaviors of children with an ADHD diagnosis. Second, secure attachment has a positive effect on the development of skills in areas in which children with ADHD demonstrate problems (e.g., attention span, impulse control). There are currently no findings on whether or not and how insecure and disorganized attachment and ADHD affect children’s emotion recognition ability. *Methods*: This is a cross-sectional study, part exploratory and part hypothesis-driven in the context of basic research. A clinical sample of 5- to 10-year-old children with an ADHD diagnosis and their parents is to be compared to a non-clinical unaffected control group. Over a period of 3 years, 80 subjects and their parents are to be recruited in each group for participation in the study. *Discussion*: This study is the first to examine links between attachment, emotion recognition ability, and ADHD. It is also the first to include not just children with ADHD but also their mothers and fathers in its design. The findings should help reduce the research gap and generate more knowledge for family interventions in the case of ADHD.

## 1. Introduction

### 1.1. ADHD, Attachment and Emotion Recognition

With an average prevalence of just over 5% [1], attention-deficit/hyperactivity disorder (ADHD) is one of the most common diagnoses in child and adolescent psychiatry on the externalising disorder spectrum [2,3]. It is highly heritable and multifactorial; multiple genes and non-inherited factors contribute to ADHD [4]. Additionally, familial aggregation of ADHD increases with increasing genetic relatedness [5], enhancing the possibility of finding psychiatric symptoms in parents of children with ADHD. The core symptoms are inattention, hyperactivity, and impulsivity. The Diagnostic and Statistical Manual of Mental Disorders, fifth edition’s (DSM-5) criteria [6] break ADHD into predominantly inattentive presentation (314.00), predominantly hyperactive/impulsive presentation (314.01) and combined presentation (314.01), while the international WHO Classification of Mental and Behavioral Disorders (ICD-10) describes an attention-deficit hyperactivity disorder (F90.0), hyperkinetic conduct disorder (F90.1), other hyperkinetic disorders (F90.8), and hyperkinetic disorder, unspecified (F90.9) [7]. For diagnosis, the core symptoms must be present to an abnormal degree and across a range of situations in family and school settings over a period of at least six months. The symptoms must also be perceived as a problem and, pursuant to ICD-10, must have been present before the child started school. In DSM-5, on the other hand, the age by which the first symptoms must have occurred has been raised to 12.

In early childhood, the child’s attachment qualities to his or her principal attachment figures develop according to how these figures deal with the child’s signals. If an attachment figure appropriately perceives everyday signals from the child, interprets them correctly, and acts promptly and suitably, Ainsworth, Bell, and Stayton [8] define this attachment figure as sensitive. The child will consider such an attachment figure in his or her internal working model (IWM) as competent, reliable, and predictable and develop a secure attachment to that figure over the course of the first year of his or her life. Insecure attachment (avoidant or ambivalent) is formed when an attachment figure responds to a child’s signals inappropriately, with the wrong timing, or unreliably. If a child is neglected, mistreated, abused or experiences the mental illness of his or her attachment figure, or is threatened or otherwise traumatized by the latter, that child will form a disorganized attachment to that attachment figure. As attachment research has shown in numerous detailed longitudinal studies [9,10,11], attachment experiences in early childhood are a strong predictor of psychosocial development all the way into adulthood. We now know that secure attachment to the primary attachment figure in the first year of a child’s life is a protective factor in his or her psychosocial development, whilst the two insecure qualities of attachment represent a less favorable developmental prognosis. Attachment disorganization, on the other hand, represents a risk factor for psychopathological development [12]. Alongside the question of whether the attachment figure succeeds in responding sensitively to the child’s signals, attachment representation on the part of the attachment figure also plays an important role in the development of the child’s attachment quality. The correlations between parental attachment representation and the child’s attachment quality, which have already been demonstrated in a range of studies, are considered as evidence for the intergenerational transmission of attachment [13,14,15]. Transmission is evidenced most commonly in secure attachment representation on the part of the mother and secure attachment quality on the part of the child, but also exists for insecure and disorganized attachment representation on the part of the mothers and the insecure and disorganized attachment of their children.

Emotional facial expressions provide necessary information for mutual social interactions. The ability to correctly decode those expressions is a key skill for the situational adjustment of social behavior. Even infants who are just a few months old are able to differentiate between happy, sad, and surprised faces and between different levels of intensity of feeling [16]. Studies on the development of emotion recognition ability have found that the ability to accurately recognize and interpret facial affects develops continuously over the course of a child’s development. The ability to recognize emotion can be measured on the basis of visual preferences. For example, studies assess how long a child remains focused on a particular optical stimulus (fixation duration) and whether the fixation duration varies depending on the type of emotion. The accuracy and speed at which a child is able to recognize emotion develops continuously throughout infancy and the pre-school years [17,18,19,20,21]. The underlying neuropsychological test procedure is based on Ekman and Friesen’s concept of the six basic emotions [22], defined as happiness, sadness, fear, anger, disgust, and surprise. Ekman and Friesen [23] developed a standardized set of stimuli for testing emotion recognition ability, in which these basic emotions are displayed in facial expressions by actors of various ages and different sexes.

### 1.2. ADHD and Attachment

Children with ADHD can represent a major challenge in everyday family life right from infancy. There are now a number of empirical indications that the temperament and vitality of the infant play a part in the etiology of ADHD, in particular in the event of a mismatch with the temperament and vitality of the primary attachment figure [24,25,26]. Prospective studies [27,28] have found that, out of around 40 different criteria relating to the child, only the quality of the parent–child interaction is a predictor for the risk of ADHD for a six-month-old infant. Such a risk was often accompanied by aspects such as overstimulating and intrusive behavior on the part of the parents, relationship issues between the parents and the child, and a lack of support for the mother. In the light of these previous findings, it is surprising that, to date, only one study [29] has explicitly presented the distribution of attachment representations in children with ADHD, and there has, so far, been no systematic study of the distribution of the attachment representation of the parents of such children. Only Kissgen et al. [30] have been able to show in a pilot study with the mothers of children with ADHD that an increasing clinical abnormality in ADHD children has a statistically very significant correlation (u = 3.78; *p* < 0.001) with a lower incidence of secure attachment representation and an increase in insecure and disorganized attachment representation on the part of the mothers.

Clear indications of a link between ADHD and attachment can be found in the previously separate research areas of ADHD, on the one hand, and attachment, on the other. Cassidy [31] has found that people with an insecure attachment are more likely to experience problems in regulating emotion and behavior. Problems of this type are also central elements of ADHD [32,33,34].

A large number of studies have also shown that secure attachment has a positive effect on the development of skill sets in areas in which children with ADHD demonstrate problems: Maslin-Cole and Spieker [35] found that securely attached infants had a higher attention span. Matas et al. [36] reported greater enthusiasm, positive affect, and greater persistence on the part of securely attached children in problem-solving situations. In pre-school children, secure attachment has been linked to the ability to respond flexibly, persistently, and imaginatively [37]. Six-year-olds with secure attachment representation have also been found to have a greater cognitive control over their impulses, a stronger focus, and more delayed gratification [38,39].

The insecurely attached, on the other hand, display more problems in interpersonal relations than the securely attached [40,41,42]. For children with ADHD, Greene et al. [43] documented greater difficulties in social functioning than in unaffected children. A more recent study of 100 children between the ages of 5 and 10 [44] was the first to focus on the relationship between ADHD and disorganized attachment representation. The authors found a clear link when they controlled for both behavioral abnormalities and executive functions (inhibitory control and working memory) and concluded that behavioral abnormalities could have a mediating effect. Control for behavioral problems is, therefore, and for the reasons outlined above, essential to any exploration of links between attachment and ADHD.

Although Salari et al. [45] found only recently that there is a separate link between attachment in middle childhood and ADHD symptoms in adolescence, we can currently say from a research perspective that there is insufficient evidence, to date, of either the effect of attachment on the later development of ADHD symptoms or the effect of ADHD symptoms on the development of attachment representation. The development of ADHD symptoms is a result of a complex combination of child, family, and environmental factors, and the specific impact of any individual factor remains unclear [46]. However, it can be shown that the development of ADHD symptoms is linked to the same factors that play a part in developing an individual attachment representation.

### 1.3. ADHD and Emotion Recognition

Adults [47] and children with ADHD have certain deficits in their ability to recognize facial expressions [48,49,50,51,52,53,54,55]. These limitations correlate with the social skills and behavioral problems of the children [51,53]. There is disagreement on the nature of those deficits and their underlying mechanisms. Some authors [48,49] conclude that the aforementioned difficulties occur with all types of emotion and stem from the impulse control and executive function deficits that have been empirically demonstrated in children with ADHD. Other researchers think that the deficits are specific to given emotions. Specific deficits in the recognition of anger and sadness [51], anger and fear [55], and fear and disgust [52] and a tendency to misinterpret various emotions as fear or sadness (“negative bias” [53]) have been described in children with ADHD. A review by Collin et al. [56] concludes, on the basis of the 14 studies reviewed, that ADHD children demonstrate significantly more problems relating to emotion recognition ability than unaffected children. However, the authors criticize the fact that the sample sizes in all studies were too small.

### 1.4. Attachment and Emotion Recognition

In childhood development, the ability to recognize emotions is linked to the memory-based ability to develop expectations of how the other person will behave if his or her face is expressing emotion (for example happiness or anger). If the other person’s behavior matches the child’s expectations, this confirms the child’s mental representations of events. This, in turn, forms the basis for the more abstract and generalized internal working model (IWM) of attachment [10,57,58,59]. Malatesta [60] concluded that a child between the ages of three and six months has to perceive, decode, and memorize an average of 32,000 emotional facial expressions from the mother alone. It is, therefore, to be assumed that the IWM includes prototypes and variations of those basic emotions that a child has experienced in interactions with his or her mother and other primary attachment figures in early childhood.

The primary attachment figures of securely attached children allow their children to experience the full spectrum of emotions. Steele et al. [61], therefore, conclude that those children, compared to those who are insecurely attached, have the best basis for emotion recognition ability. Unlike securely attached children, disorganized children have been mistreated, abused or neglected, or have experienced other forms of trauma. In order to anticipate the behavior of their negative attachment figures, children have to become experts at recognizing the expressions worn by those attachment figures before they once again place the child in danger or indeed traumatize him or her. It would, therefore, appear that these children are better at recognizing negative emotions than positive emotions. From an attachment research perspective, Steele et al. [61] investigated whether attachment quality in early childhood is a predictor of emotion recognition ability at the ages of 6 and 11. In their underlying longitudinal study, they found that the quality of the child–mother attachment at 12 months was a predictor of the child’s emotion recognition ability at the ages of 6 and 11. At these ages, the children who had been securely attached at 12 months did statistically significant better than those who had had an insecure or disorganized attachment at 12 months.

### 1.5. Objectives

Overall, it can be concluded in the light of the basic theory outlined and research findings, to date, that there are significant research desiderata regarding the links between ADHD and attachment, ADHD and emotion recognition, and attachment and emotion recognition. The study seeks to make a significant contribution to those research desiderata in order to better understand the situation of children with ADHD and their parents and generate more knowledge for family dynamics in the case of ADHD.

### 1.6. Key Questions

What is the distribution of attachment representations of children with ADHD and their parents in comparison to clinically not affected children and their parents?

- Does the intergenerational transmission of attachment in the families of children with an ADHD diagnosis differ from that in families of clinically not affected children?
- Are there links between ADHD and emotion recognition ability?
- Are there links between the attachment representation of children and their emotion recognition ability?
- Are there complex links between the attachment representation of parents, the attachment representation of children, ADHD diagnoses, and emotion recognition ability?

## 2. Materials and Methods

This is a cross-sectional study in the context of basic research, part exploratory and part hypothesis-driven. A clinical sample of children with an ADHD diagnosis and their parents is compared to a clinical unaffected control sample.

### 2.1. Participants

The clinical sample comprises 5–10-year-old ADHD children (*n* = 80) who are being treated as in-patients or day-care patients at the Department of Child and Adolescent Psychiatry, Psychosomatics, and Psychotherapy at the University Clinic of Cologne/Germany. The study also includes the children’s parents. The non-clinical, parallelized control sample (*n* = 80) are to be recruited from schools in Siegen, Bonn, and Cologne.

Inclusion criteria:

○ Children in the clinical group: a confirmed clinical diagnosis of ADHD in accordance with ICD-10 (F90.0, F90.1) or DSM-5 (314.00, 314.01);
○ Children in the control group: no infant or adolescent psychiatric diagnosis (acute or lifetime);
○ Written consent to participate in the study after an explanation of the study to the parents and the child;
○ Ages: 5–10;
○ A good command of the German language.

Exclusion criteria:

○ IQ < 70;
○ Additional child or adolescent psychiatric diagnosis;
○ Serious neurological or somatic diseases;
○ Other disorders relating to perception, cognition, or affect that would impede or prevent a neuropsychological assessment;
○ Persons taking medication affecting the CNS (with the exception of methylphenidate);
○ Single-parent family.

### 2.2. Design

The study is to be completed within a period of 3 years from the start of data collection. The aim is to complete data collection as far as possible after the first 30 months. The final 6 months are for data processing, statistical analyses, and reporting. Statistical analysis is to be carried out by the Chair of Developmental Science and Special Education at the University of Siegen.

Inpatients will be diagnosed within the first three weeks of treatment. The time for conducting the attachment inventories is c. 40 min per person (child, mother, and father). A time of 30 min has been estimated for the neuropsychological assessment of the children. Children who are being treated with methylphenidate will be assessed in the morning before taking their medication (half-life of methylphenidate: 3–4 h). The control group is being recruited from participating schools in Siegen, Bonn, and Cologne. The time estimated for diagnosis and neuropsychological testing corresponds to that for the test group. Sociodemographic data will also be collected, and intelligence testing carried out for the child at the assessment appointment. The time for collecting all data, including breaks for the families, is c. 3 h. As an incentive, all test persons receive a cinema voucher worth EUR 15.

### 2.3. Instruments

All questionnaires have been validated or have been used in other studies and published in the literature.

#### 2.3.1. Sociodemographics

Data will be collected on nationality, socioeconomic status, family circumstances and the school career of the subjects. These details are to be supplemented with questions on the risk exposure of the family.

#### 2.3.2. Intelligence

The Wechsler Intelligence Scale for Children-IV (WISC-IV) [62] is to be used as the standard inventory. Testing is not required if there are previous findings from outpatient treatment available that are no more than six months old.

#### 2.3.3. Clinical Diagnosis

The initial diagnosis is by the attending child and adolescent psychiatrists. Diagnostic checklists (DCL) and external assessment questionnaires (FBB) from the ICD-10 and DSM-IV classification of mental and behavioral disorders in children and adolescents (DI-SYPS-KJ) [63] will also be used/completed for each subject for inpatient assessment of the inclusion and exclusion criteria. The psychometric quality of these inventories has been proven:

- Diagnostic checklist for hyperkinetic disorders (DCL-HKS)
- Diagnostic checklist for anxiety disorders (DCL-ANG)
- Diagnostic checklist for depressive disorders (DCL-DES)
- External assessment questionnaire for hyperkinetic disorders (FBB-HKS)
- External assessment questionnaire anxiety disorders (FBB-ANG)
- External assessment questionnaire for depressive disorders (FBB-DES)

The Child Behavior Checklist (CBCL/4-18) will be used to check externalizing and internalizing behavioral abnormalities [64].

#### 2.3.4. Attachment Representation

Determining attachment representation at the various different ages is methodologically highly complex as it involves age-specific inventories requiring special training. All researchers on the study have successfully completed the required training at certified institutions. The attachment inventories conducted will be evaluated exclusively by reliable colleagues from other institutes who are not involved in the study itself.

Attachment is to be diagnosed for participating children using the Geschichtenergänzungsverfahren (GEV-B) procedure [65]. In terms of content and implementation, this procedure is the German adaptation of the attachment story completion task (ASCT) [66]. Participating children are told the beginnings of stories relevant for attachment, which are played out with small dolls. The children must then complete the stories. Alongside the evaluation of content, how a child deals with the attachment issues presented is recorded in a video and can then be classified to allow conclusions on attachment to be drawn. The inventory allows the categorization of the attachment representation of participating subjects and also provides an attachment score that can be used for statistical calculations.

The adult attachment projective (AAP) [67] is to be conducted with the participating adults. In this process, subjects are shown cards depicting scenes designed to activate the attachment behavioral system. Subjects are asked to tell a story for each card: what is happening in the scene shown, what led to that situation, what are the people thinking and feeling, and what will happen next. The interview will be recorded, transcribed, and then classified in terms of content, discourse, and defensive processes by reliable evaluators. This procedure makes it possible to determine the attachment representation of the subjects.

#### 2.3.5. Emotion Recognition

The study uses the Diagnostic Analysis of Nonverbal Accuracy 2 (DANVA-2) [68]. This is a standardized, validated tool that is widely used in child and adolescent psychiatric research, in particular for young age groups. DANVA-2 uses children’s faces as well as adult faces in addition to the standard pictures of facial affect from Ekman and Friesen [23]. Emotions are also depicted in varying levels of intensity so that it is not only possible to draw a general conclusion about whether and how well they are able to recognize basic emotions but also to detect strengths or weaknesses in the recognition of individual emotions (low v. high intensity).

### 2.4. Sample Size and Power Calculation

As the questions outlined have never before been investigated at this level of complexity, the literature only provides a limited indication of the scale of effects to be expected. The optimum sample size is calculated on the basis of the most complex question, the link between attachment representation and emotion recognition ability in children with ADHD. Expected effect sizes can most usefully be calculated on the basis of literature on the links between ADHD and emotion recognition. These effects range from a partial eta^2^ = 0.04 in the study by Kats-Gold et al. [53] to eta^2^ = 0.81 in the study by Cadesky et al. [49], i.e., the spread is very wide. On this basis, and in the light of the fact that small partial effect sizes to be estimated using regression analysis models would require an unrealistically high number of subjects, this study seeks to establish moderate effects at least. The following parameters were also considered: fixed-effects regression model with a maximum of six predictors and one criterion, alpha error level of 5%, and a beta error of no more than 10% (corresponds to a power of 0.9). The sample size calculated in line with these specifications is *n* = 123 (GPower 3.1.7, Heinrich Heine University Duesseldorf) [69]. As the study of each subject also involves the study of both parents, it is assumed that data will be incomplete in c. 25% of cases. The final sample size is therefore 160 subjects.

### 2.5. Data Analysis

Data is to be collected using standardized and tested instruments. A folder of findings for each participant in the study is to provide quick and easy access to the relevant findings. Data is primarily to be evaluated using the software package SPSS 26 (SPSS: an IBM company, Chicago, IL, USA) at the Chair of Developmental Science and Special Education at the University of Siegen. To this end, the raw test results will be transformed into standard values (*z*-values) where necessary, using the average and standard deviation from the non-clinical sample. The hypotheses on difference to be assessed will be explored using group comparisons. Parametric procedures will be used where data dignity allows; in other cases, appropriate non-parametric procedures will be employed. Correlation hypotheses are to be tested using AN(C)OVAS or multi-factor regression analyses. To improve the classification and comparability of results, the appropriate effect sizes for the procedures used are also to be calculated.

## 3. Discussion

This article presents the design of a study that is, for the first time, to examine links between attachment, emotion recognition ability, and ADHD in 5- to 10-year-old children and their parents. Over a period of 3 years, 80 children and their mothers and fathers are to be studied in the clinical group (children with an ADHD diagnosis) and the same number in the control group (children with no clinical diagnosis).

One strength of the study is the sample size. If the intended case number is achieved, a total of 480 persons will be studied. Another innovation is that the study includes not just children with ADHD but also their mothers and fathers in its design. For the first time, it will, therefore, be possible to describe family dynamics from a range of perspectives on the basis of data and evaluate those dynamics in comparison with unaffected families. On the significance of fathers in particular, no data are available, to date, in the area outlined. Another strength is that the study uses questionnaires to check for other child and adolescent psychiatric clinical pictures in addition to ADHD.

The limitations include the expected proportions of the different sexes. In clinical studies, it can be assumed the ratio of girls to boys will be 1:9. It will not be possible either to influence the proportion of F90.0 (attention-deficit hyperactivity disorder) to F90.1 (hyperkinetic conduct disorder) diagnoses in accordance with ICD-10. This is significant, as it is to be assumed that these are two distinctive clinical pictures that should not be combined in one group for statistical purposes [70,71]. Whilst it is to be assumed that all social backgrounds will be represented in the test group, this is less likely for the control group. Experience has shown that conventional middle-class families are more likely to take part in control groups whilst low-income families rarely do.

## 4. Conclusions

To conclude, the study coordinators hope, on the one hand, to reduce the gap in the research in the areas outlined. They also believe that the findings obtained will contribute to advisory and psychotherapeutic work with children with an ADHD diagnosis and their parents.

## Data Availability

The datasets used and/or analyzed during the current study are available from the corresponding author on reasonable request.

## Trial Registration

German Clinical Trials Register (DRKS): DRKS00007812; Date registered: January 27, 2020; Retrospectively registered, https://www.drks.de/drks_web/navigate.do?navigationId=trial.HTML&TRIAL_ID=DRKS00007812 (accessed on 22 January 2021).
